# Instagram as a social media tool about orthognathic surgery

**DOI:** 10.15171/hpp.2019.44

**Published:** 2019-10-24

**Authors:** S Kutalmış Buyuk, Tugce Imamoglu

**Affiliations:** Department of Orthodontics, Faculty of Dentistry, Ordu University, Ordu, Turkey

**Keywords:** Social media, Orthognathic surgery, Instagram, Patient perspective

## Abstract

**Background:** The aim of this short communication was to evaluate the quality and content of the Instagram posts about orthognathic surgery.

**Methods:** Two hashtags #jawsurgery and #orthognathicsurgery were searched on Instagramin this retrospective Instagram post analysis study. Selected 100 posts for each hashtag were analyzed for type, total number of likes, comments, purpose and source.

**Results:** Most of the posts about #jawsurgery were uploaded by patients (56.1%) who shared their personal experience. Most of the posts about #orthognathicsurgery also were uploaded by clinics (49.1%). Most of posts (39.6%) provided information about orthognathic surgery in #orthognaticsurgery. Posts generally involved by patient’s experience (49.1%), followed by patient information (28.1%), and advertisement (22.8%) in #jawsurgery.

**Conclusion:** Patients should be educated by orthodontists and maxillofacial surgeons about Instagram platform that it is not an adequate or accurate source of information about orthognathic surgery in its current form.

## Introduction

Internet is now an indispensable part of human life and a main source of information. Patients view the Internet as a valuable source of health information and it affects their relationship with healthcare professionals.^1,2^ Despite the potential benefits of finding health information on the Internet, the possibility of misinformation becomes a serious issue. The dissemination of incorrect information through social media accounts is an important problem, as anyone can share anything on these media without the supervision of experts or moderators.

Dental malocclusions can be corrected with orthodontic treatment; however, cranio-facial deformities at the skeletal level require orthognathic surgery. Orthognathic surgery operations are now a routine procedure for surgeons, but patients view it as a series of complex procedures that require larger, longer, and unknown processes.^3,4^ Therefore, supporting the patient with a psychological influence and adequate information is of crucial importance during the stage of decision making. Adult patients use internet as a source of information as they try to decide about orthognathic surgery. Some patients usually receive information from their peers who have orthognathic surgery before the procedure, while others receive detailed information directly from the maxillo-facial surgeon.^5,6^ Both approaches have their advantages and disadvantages, and people can now have quick and easy access to information about any subject in the field of medicine or dentistry through social media.

Instagram is a free photograph and video sharing application also serving as a social media platform. It was founded in October 2010. It acquired a huge user base in a very short time and currently ranks the 15th in the list of most visited web sites in the world. Considering it has more than one billion users, the average daily visitor spends 5 minutes 42 seconds on the on-site, and more than 100 million photographs or videos are uploaded daily.^6-8^ Instagram hosts all types of accounts in all fields imaginable and as for the fields of dentistry and medicine, many posts are performed by doctors, clinics, and patients with a variety of videos or photographs covering personal experience or suggestions. Also, patients are seeking aesthetic surgery to improve their appearance on Instagram, and other social media platforms.^9^ However, all this information is uploaded without any type of expert review; therefore, its accuracy and reliability are not guaranteed.

Some studies were performed on the relationship between social media and orthognathic surgery.^6,10^ However, no study was found that investigated the information about orthognathic surgery posts on Instagram. The aim of this study is to evaluate the quality and content of the Instagram posts about the orthognathic surgery.

## Materials and Methods

Public posts on Instagram (www.instagram.com) with the hashtags “#jawsurgery” and “#orthognathicsurgery” were screened manually by one of the authors (T.I.) on March 29, 2019. This study was planned as a cross-sectional study. The hashtags were designed based on Coleman et al,^11^ who evaluated the two hashtags most frequently used by patients and laypeople in their article on the influence of social media. This study did not require the approval of the ethics committee, since it contains only public data. The total number of posts per hashtag was recorded and 100 posts were selected for each hashtag in order of date and relevance. The URL of each post was recorded. Furthermore, the total number of ‘likes’ and comments for the selected posts was categorized, as well as the type of posts (video, photograph, text), the date it was posted, its purpose. Also, each post was recorded together with the information whether they were an orthodontist, surgeon, clinic or patient. Posts that were duplicates, or not related to orthognathic surgery were excluded from the study with the consensus of the authors. All recorded URLs of each post were analyzed 5 weeks later, and deleted or inaccessible posts removed. Consequently, 57 posts about #jawsurgery and 53 posts about #orthognathicsurgery were evaluated in this study.

Data was performed by a researcher of this study in a Microsoft Excel (Microsoft, Inc, Redmond, WA) spread sheet. Descriptive statistics were performed for posts containing type of post, number of likes, number of comments, source of post, and purpose of posts. Statistical analysis was done with IBM SPSS Statistics statistical software (version 25, IBM SPSS Statistics, Armonk, USA). Kruskal–Wallis test was performed to determine the differences between sources of posts. The significance level was set at 5%.

## Results

A total of 73 584 posts were made with the #jawsurgery (50 806) and #orthognathicsurgery (22 778), mostly related to orthognathic surgery used by laypeople. Table 1 shows the number of likes, number of comments, and other descriptive characteristics of the posts.

The posts with the #jawsurgery included 53 photographs, 3 videos, and 1 text. As for their sources, 32 of them were posted by patients, 16 by clinics, 7 by orthodontists, and 2 by surgeons. The assessment of the purpose showed that 34 posts were about sharing personal experience, 15 were for advertisement, and 11 were informative about the orthognathic surgery.

The posts about the #orthognathicsurgery included 43 photographs, 9 videos and 1 text. Source investigation showed that 12 of them were uploaded by surgeons, 13 by patients, 26 by clinics, and 2 by orthodontists. While 21 posts provided information about orthognathic surgery, 15 included accounts of personal experiences about orthognathic surgery, and the remaining 17 were advertisement.

The languages of the posts about #jawsurgery are as follows: English (52 posts), Spanish (3 posts), Polish (1 post), and Korean (1 post). The languages of the posts about #orthognathicsurgery are as follows: English (38 posts), Portuguese (6 posts), Spanish (5 posts), Turkish (2 post), Italian (1 post), and Greek (1 post).

Posts about the #orthognaticsurgery that shared by orthodontists or surgeons were had significantly more mean likes than shared by patients and clinics (*P*= 0.024) (Table 2). There is no statistically significant #jawsurgery posts like and comments among the groups (*P* > 0.05)

## Discussion

As the power of social media increases every day, millions of people rely heavily on their social media accounts for information to the extent of seeking answers on the Internet first for their medical or dental problems instead of consulting with doctors. The Instagram application is one of the social media platforms patients prefer as it provides easy access to information, videos, and photographs about orthognathic surgery as well as other health-related topics. The number of photographs or videos uploaded daily is over 100 million and patients have quick access to photographs, comments, or videos about orthognathic surgery.^7^ In this study, we aimed to investigate the content of posts shared about orthognathic surgery on Instagram.

Patients may shy away from asking as many questions as they would like to interview with their doctors. This is one of the factors that make them turn to the Internet, and social media as a source of information.^9^ However, the reliability and accuracy of the photographs and videos shared on Instagram are controversial because it is a ‘social’ platform, everyone shares their opinion. Most videos on social media about orthognathic surgery are posted by patients or clinical advertisement agencies and small number of them are coming from the field specialists, such as maxillofacial surgeons or orthodontists.^10^ Our study also showed that many of the Instagram posts about orthognathic surgery were shared by laypeople or patients. Also, the photographs and videos shared never undergo any review or evaluation process and users can choose any hashtags they want, which leaves the posts with little credibility and reliability.

Hegarty et al^10^ evaluated the orthognathic surgery videos with the highest number of viewers on another social media platform YouTube^TM^. They concluded that 55.83% of the videos presented low quality information and those with perfectly informative content made up a percentage as small as 9.17%. Moreover, they noted that most videos were about the personal experiences of patients, thus contained a high degree of bias. The conclusion of the study was that YouTube^TM^ videos on orthognathic surgery were sub-standard and that patients should be recommended to view it with caution. Our study supports this conclusion in that most of the posts on Instagram about orthognathic surgery were shared by patients and poorly informative.

The former system of face-to-face discussion with the doctor is thus replaced with a convenient, free, and easily accessible alternative method social media on Internet. Watts et al^12^ investigated Twitter posts under #jawsurgery dividing them into 3 main categories: pre-operative engagement, post-operative difficulties, and post-treatment satisfaction. The most commonly used tweets were ‘recovery,’ ‘eat,’ ‘braces,’ ‘liquid diet,’ ‘swollen,’ and ‘pain.’ As a result of the study, clinicians were advised to warn their patients who needed orthognathic surgery about social media and to offer comprehensive guidance about the treatment procedure. In our study, we have the similar conclusion that clinicians should provide guidance to their patients explaining to them the need to be careful about the source and purpose of the Instagram posts about orthognathic surgery.

Another social media study performed by Coleman et al^11^ investigated how patients used social media in relation to orthognathic surgery. The patients were asked to fill a questionnaire containing 15 questions. It was observed that 87% of the participants had less anxiety after researching orthognathic surgery in social media. Most of the orthognathic surgery patients surveyed indicated their most frequent search terms as ‘jaw surgery’ and ‘orthognathic.’ In our study, we also searched the #jawsurgery and #orthognathicsurgery as these two appeared to be the most frequent search terms used by laypeople on Instagram.

To better educate individuals about the mentioned surgical procedures on social media, one recommendation can be that authoritative bodies such as associations of orthodontists and maxillofacial surgeons promote the advantages of orthognathic surgery by reposting good quality content published by clinician members.

One of the most important limitations of this study is that it was based on the evaluation of most recent posts performed in a single time range on the Instagram platform. Another limitation is that the data changes continuously on Instagram platform.

## Conclusion

Individuals should be educated by orthodontists and maxillofacial surgeons about Instagram platform that it is not an adequate or accurate source of information about orthognathic surgery in its current form. Further prospective and unbiassed studies about the orthognathic surgery in different social media platforms should be performed.

## Ethical approval

This study did not require the approval of the ethics committee, since it contains only public data.

## Competing interests

The authors declare that they have no competing interests.

## Funding

None.

## Authors’ contributions

SKB and TI collected the data. SKB and TI were involved in the conception of the study, performed the analyses, and drafted the manuscript. SKB designed the methods and wrote the conclusion. All authors reviewed and edited the manuscript and approved the final version.

**Table 1:** Comparison of Instagram characteristics of posts tagged with #jawsurgery and #orthognathicsurgery

		**#jawsurgery(57)**	**#orthognathicsurgery(53)**
Post type	Photograph	53	43
	Video	3	9
	Text	1	1
Number of likes	0-50	39	28
	51-100	12	11
	>100	6	14
Number of comments	0-5	49	42
	6-10	6	4
	>10	2	7
Source	Surgeon	2	12
	Patient	32	13
	Clinic	16	26
	Orthodontist	7	2
Purpose	Surgical information	16	21
	Patient experience	28	15
	Advertisement	13	17

**Table 2:** Comparison of hashtag number of likes and comments according to source of posts

	**#jawsurgery**		**#orthognathicsurgery**	
	**Like**	**Comment**	**Like**	**Comment**
	**Median (Min-Max)**	**Median (Min-Max)**	**Median (Min-Max)**	**Median (Min-Max)**
Orthodontist/Surgeon	27 (13-180)	2 (0-4)	80.50 (25-1041)	2.50 (0-147)
Patient	34 (0-312)	2 (0-19)	23 (0-222)	2 (0-27)
Clinic	29.50 (1-530)	1.50 (0-16)	31.50 (5-532)	0 (0-20)
*P* ^a^	0.887	0.871	0.024	0.066

Min; Minimum, Max; Maximum; ^a^Results of Kruskal-Wallis test.

## References

[1] McMullan M (2006). Patients using the Internet to obtain health information: how this affects the patient-health professional relationship. Patient Educ Couns.

[2] Eysenbach G, Köhler C (2004). Health-related searches on the Internet. JAMA.

[3] Øland J, Jensen J, Melsen B (2010). Factors of importance for the functional outcome in orthognathic surgery patients: a prospective study of 118 patients. J Oral Maxillofac Surg.

[4] Hunt OT, Johnston CD, Hepper PG, Burden DJ (2001). The psychosocial impact of orthognathic surgery: a systematic review. Am J Orthod Dentofacial Orthop.

[5] Flanary CM, Barnwell GM Jr, Alexander JM (1985). Patient perceptions of orthognathic surgery. Am J Orthod.

[6] Larsen MK, Thygesen TH (2016). Orthognathic surgery: outcome in a Facebook Group. J Craniofac Surg.

[7] Alexa. youtube.com Traffic Statistics. http://www.alexa.com/siteinfo/instagram. Accessed 12 May 2019.

[8] Aslam S. Instagram by the numbers: stats, demographics & fun facts. Available from: https://www.omnicoreagency.com/instagram-statistics. Accessed 12 May 2019.

[9] Dorfman RG, Vaca EE, Mahmood E, Fine NA, Schierle CF (2018). Plastic Surgery-Related Hashtag Utilization on Instagram: Implications for Education and Marketing. Aesthet Surg J.

[10] Hegarty E, Campbell C, Grammatopoulos E, DiBiase AT, Sherriff M, Cobourne MT (2017). YouTube as an information resource for orthognathic surgery. J Orthod.

[11] Coleman O, Walker TWM, Kerai A, van der Valk R, Thomas SJ (2018). #JawSurgery: analysis of social media use in orthognathic surgery patients. Br Dent J.

[12] Watts GD, Christou P, Antonarakis GS (2018). Experiences of individuals concerning combined orthodontic and orthognathic surgical treatment: a qualitative Twitter analysis. Med Princ Pract.

